# Expression Profile and Prognostic Values of *HOXA* Family Members in Laryngeal Squamous Cell Cancer

**DOI:** 10.3389/fonc.2020.00368

**Published:** 2020-03-31

**Authors:** Jinyun Li, Meng Ye, Chongchang Zhou

**Affiliations:** ^1^Department of Oncology and Hematology, The Affiliated Hospital of Medical School of Ningbo University, Ningbo, China; ^2^Department of Otorhinolaryngology Head and Neck Surgery, Ningbo Medical Center Lihuili Hospital, Ningbo, China

**Keywords:** *HOXA* family, TCGA, prognosis, GSEA, LSCC

## Abstract

The homeobox A cluster (*HOXA*) gene family, comprising 11 members, is involved in a wide spectrum of biological functions in human cancers. However, there is little research on the expression profile and prognostic values of *HOXA* genes in laryngeal squamous cell cancer (LSCC). Based on updated public resources and integrative bioinformatics analysis, we assessed the expression profile and prognostic values of the *HOXA* family members. Expression and methylation data on *HOXA* family members were obtained from The Cancer Genome Atlas (TCGA). The prognostic values of *HOXA* members and clinical features were identified. A gene set enrichment analysis (GSEA) was conducted to explore the mechanism underlying the involvement of *HOXA* members in LSCC. The associations between tumor immune infiltrating cells (TIICs) and the *HOXA* family members were evaluated using the Tumor Immune Estimation Resource (TIMER) database. *HOXA2* and *HOXA4* were downregulated and *HOXA7* and *HOXA9*–*13* were upregulated in LSCC. Upregulation of *HOXA10, HOXA11*, and *HOXA13*, along with two clinical characteristics (M stage and gender), were associated with a poor LSCC prognosis based on the results of univariate and multivariate Cox proportional hazards regression analyses. Although there were no significant correlations between TIICs and *HOXA* members, the GSEA results indicated that *HOXA* members participate in multiple biological processes underlying tumorigenesis. This study comprehensively analyzed the *HOXA* members, providing insights for further investigation of the *HOXA* family members as potential targets in LSCC.

## Introduction

Laryngeal cancer is one of the most common malignancies in the head and neck region, and laryngeal squamous cell cancer (LSCC) accounts for more than 95% of cases (1). Despite progress regarding comprehensive therapeutic strategies to treat LSCC, the prognosis of LSCC remains unsatisfactory, as 30–40% of patients die within 5 years of diagnosis with advanced LSCC (2). Identification of reliable biomarkers for LSCC prognosis could facilitate individualized treatment.

The *HOX* gene family is one of the families of homeobox genes that function as developmental regulatory genes (3). In mammals, there are 39 HOX genes in four gene clusters named *HOXA, HOXB, HOXC*, and *HOXD* (4). The *HOXA* cluster comprises 11 genes (including *HOXA1, HOXA2, HOXA3, HOXA4, HOXA5, HOXA6, HOXA7, HOXA9, HOXA10, HOXA11*, and *HOXA13*), which encode proteins that contain the DNA-binding homeobox motif (5). The molecular functions of the *HOXA* family cover a wide spectrum of biological processes, including differentiation, proliferation, migration and cell death. A substantial body of scientific evidence indicates that the expression of particular *HOXA* genes is dysregulated in certain types of carcinomas, which contributes to carcinogenesis (6–10). For instance, *HOXA1* mRNA and protein expression is upregulated in breast cancer, and forced expression of *HOXA1* in human breast cancer cells resulted in increased cell proliferation and doxorubicin resistance (11, 12). Aberrantly expressed *HOXA6* and *HOXA13* were also observed in breast cancer (13). In colorectal cancer, *HOXA13* was expressed more in normal colons than in malignant colons, and it was more highly expressed on the left side of the normal colon compared to the right side, indicating that differential *HOXA* gene expression occurs in an organized manner (10). Additionally, several studies have reported that *HOXA9* and *HOXA10* can serve as predictive biomarkers of poor survival in glioblastoma multiforme (GBM) (14–16).

Collectively, the differential expression and prognostic values of the *HOXA* family members have been noticed in various types of cancers. Studying the differential expression of *HOXA* genes in LSCC provides an opportunity to advance our understanding of LSCC development and to develop new therapeutic agents. In this study, based on updated public resources and integrative bioinformatics analysis, the expression profile and prognostic values of the *HOXA* family members were comprehensively assessed.

## Materials and Methods

### The Cancer Genome Atlas (TCGA) mRNA Expression Data of the *HOXA* Family

The TCGA program was conducted by the National Cancer Institute and National Human Genome Research Institute to molecularly characterize over 20,000 primary cancer samples and matched normal samples spanning 33 cancer types, including 528 cases of primary head and neck squamous carcinoma (HNSC), two cases of metastatic HNSC and 74 adjacent normal control samples. A total of 111 cases of laryngeal squamous cell cancer (LSCC) and 12 normal controls were included in the current study, after matching clinical parameters (including gender, age, smoking history, alcohol consumption, tumor (T) stage, node (N) stage, metastasis (M) stage, clinical stage and primary cancer sites). Subsequently, we used the Genomic Data Commons (GDC) Data Transfer Tool recommended by TCGA to download high-throughput sequencing (HTSeq) Fragments Per Kilobase of transcript per Million mapped reads (FPKM) data on the *HOXA* family.

### Comparison of the mRNA Expression of the HOXA Family in LSCC and Normal Tissues

Using Perl 5.26 software, the mRNA expression levels of the *HOXA* family were obtained from the HTSeq level 3 data on genome mRNA expression. The differential expression of the *HOXA* family in LSCC tissues compared to normal tissues was analyzed utilized the *limma* package in R 3.6.0 software. The results were visualized using the *pheatmap* package.

### Correlation Between mRNA Expression and Methylation of the *HOXA* Family in LSCC

We used the GDC Data Transfer Tool recommended by TCGA to download data from Illumina HumanMethylation 450K on the methylation levels of cg sites in the gene promoter regions of differentially expressed *HOXA* members in LSCC tissues. Thereafter, we utilized the *corrplot* package to further explore the correlation between methylation and *HOXA* expression in LSCC. The information on cg sites from Illumina HumanMethylation 450K were annotated using the annotation file from the official Illumina website (https://support.illumina.com/downloads/~infinium_humanmethylation450_product_files.html).

### Survival Analysis of *HOXA* Members in LSCC

The prognostic values of the *HOXA* members were investigated using the following two steps: (1) the associations between *HOXA* members, as well as each clinical parameter, and overall survival among LSCC patients were assessed using univariate Cox proportional hazards regression analyses and (2) using multivariate Cox proportional hazards regression analysis, the independent prognostic values of the *HOXA* members were then obtained by controlling for the significant clinical parameters from step 1. All the analyses were performed using the *survival* package in R 3.6.0 software.

### Associations Between Tumor Immune Infiltrating Cells (TIICs) and the *HOXA* Family Using the Tumor Immune Estimation Resource (TIMER) Database

Tumor cells and TIICs interact through multiple genes and pathways during cancer progression. To explore the correlations between TIICs and *HOXA* members, we utilized the TIMER platform (https://cistrome.shinyapps.io/timer/), which is an online tool for assessing the specific gene(s) associated with TIICs (17). In TIMER, the TIICs include B-cells, CD4^+^ T-cells, CD8^+^ T-cells, dendritic cells, macrophages and neutrophils.

### Gene Set Enrichment Analysis (GSEA)

To evaluate the potential mechanism underlying the involvement of *HOXA* members in the carcinogenesis of LSCC, we performed GSEA (version 4.0.1; http://software.broadinstitute.org/gsea/index.jsp) to identify the to identify the pathways related to the differential *HOXA* expression in the TCGA LSCC tissues (18). The annotated gene set file c2.cp.kegg.v7.0.symbols.gmt (from the Msig database) was used as the reference. GSEA was performed using a random combination number of 1,000 permutations and a false discovery rate (FDR) < 0.05 to identify the significantly enriched pathways.

### Statistical Analysis

The HTSeq FPKM mRNA data from the TCGA database was handled using Perl 5.26 software. The *limma* package was applied to analyze the expression of *HOXA* members in LSCC tissues, the *corrplot* package was used for the correlation between methylation and expression of *HOXA* members, the *survival* package was used for the analysis of prognostic values, the *ggplot* package was used to plot forest plots related to the multivariate Cox proportional hazards regression analysis.

## Results

### Expression Status of *HOXA* Members in LSCC Tissues

**Figure 1:**
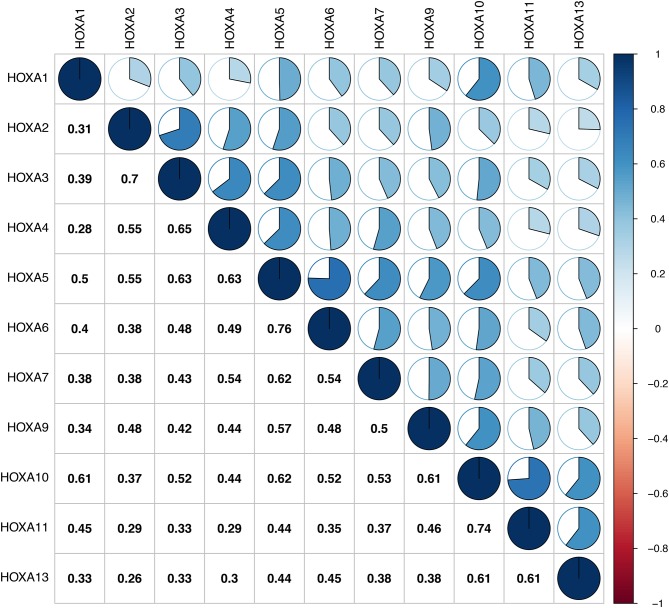
Associations between *HOXA* family members.

First of all, the mRNA expression data on *HOXA* members (*HOXA1*–*13*) from 111 LSCC samples and 12 normal control samples, which originated from TCGA, were obtained using Perl software. Pearson's correlation of *HOXA* family genes were calculated and used to assess whether these genes were correlated with each other using the *corrplot* package. As shown in Figure 1, the *HOXA* family genes were correlated to a significant degree.

**Figure 2:**
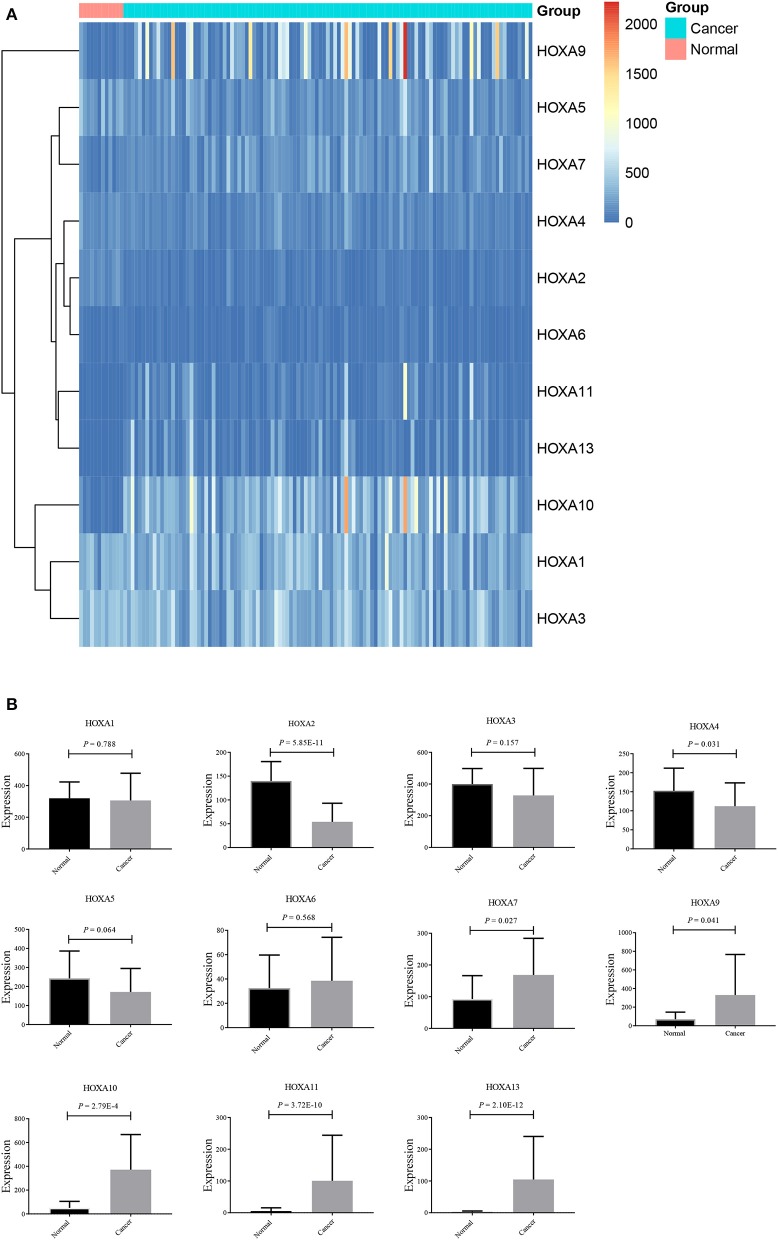
Expression profile of *HOXA* members in LSCC represented by a heatmap **(A)**, and histograms **(B)**.

Thereafter, the differentially expressed *HOXA* members were analyzed using the *limma* package and visualized using the *pheatmap* package, as shown in Figure 2A. As shown in Figure 2B, *HOXA2* and *HOXA4* were significantly downregulated in LSCC tissues compared to control tissues, while *HOXA7, HOXA9, HOXA10, HOXA11*, and *HOXA13* were significantly upregulated in LSCC tissues. There were no significant differences in *HOXA1, HOXA3, HOXA5*, and *HOXA6* expression between LSCC and control tissues.

### Correlation of HOXA Expression and Methylation in LSCC

**Figure 3:**
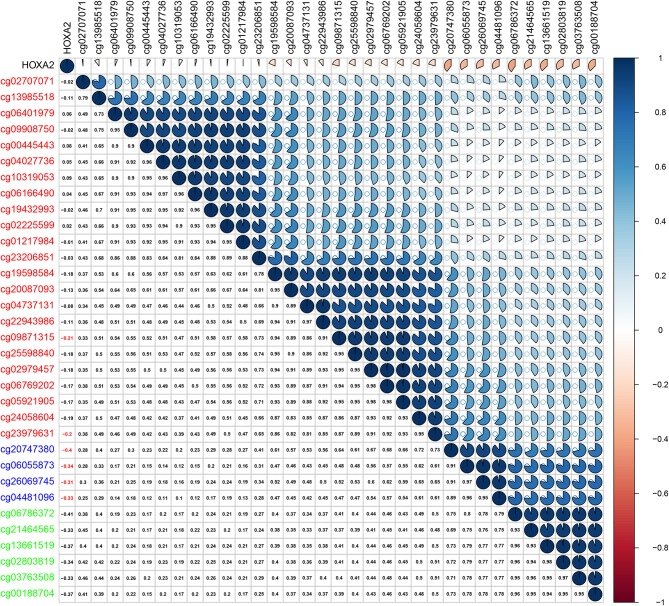
Pearson's correlation between methylation levels and expression of *HOXA2*.

Methylation of gene promoter regions is one of the most common mechanisms that influences gene expression during the progression of human cancer. We identified seven differentially expressed *HOXA* members in LSCC (downregulated *HOXA2* and *HOXA4* and upregulated *HOXA7, HOXA9, HOXA10, HOXA11*, and *HOXA13*). The Pearson's correlation results showed that six of seven differentially expressed *HOXA* members (including *HOXA4, HOXA7, HOXA9, HOXA10, HOXA11*, and *HOXA13*) was negative associated with methylation level (Figure S1), and only five of the 32 assessed CG sites in the promoter region of *HOXA2* exhibited negative correlation with *HOXA2* expression in LSCC (Figure 3). These results indicated the inverse correlation between expression and methylation level of *HOXA* members in LSCC.

### Prognostic Values of *HOXA* Members in LSCC

**Figure 4:**
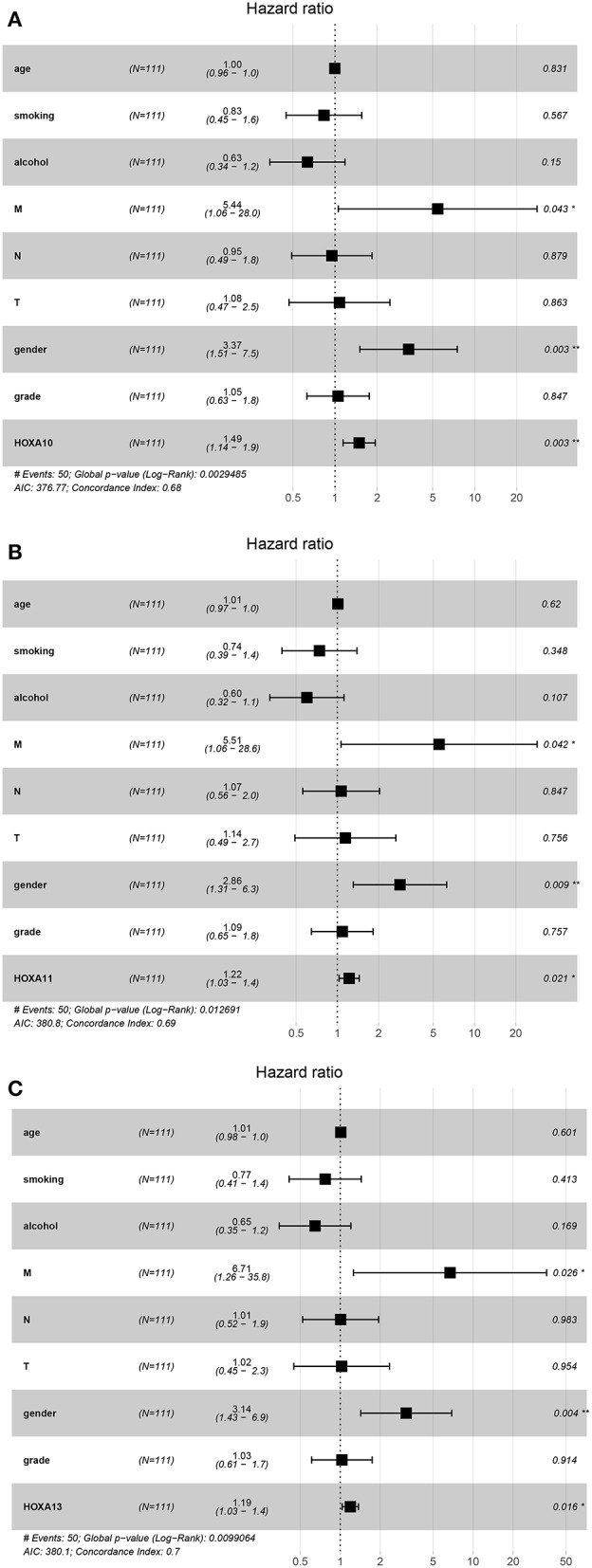
Forest plots of the results of multivariate Cox regression analyses of significant prognostic factors: *HOXA10* **(A)**, *HOXA11* **(B)**, and *HOXA13* **(C)**. *stands for *P* < 0.05; **stands for *P* < 0.01.

Subsequently, the prognostic values of *HOXA* members were analyzed. First, the predictive capabilities of differentially expressed *HOXA* members (*HOXA2, HOXA4, HOXA7, HOXA9, HOXA10, HOXA11*, and *HOXA13*) and clinical features were assessed by univariate Cox proportional hazards regression analyses. The results showed that the expression of three *HOXA* members (*HOXA10, HOXA11*, and *HOXA13*) and two clinical features (M stage and male) were associated with poor outcome of LSCC patients (hazard ratio [HR] for *HOXA10*: 1.379 (1.081–1.759); HR for *HOXA11*: 1.179 (1.000–1.391); HR for *HOXA13*: 1.129 (0.999–1.277); HR for M stage: 8.225 (1.901–35.594); and HR for male: 3.367 [1.708–6.639]) (Table 1). Second, the independent prognostic values of *HOXA10, HOXA11*, and *HOXA13* were assessed using multivariate Cox proportional hazards regression analysis to control for the prognostic effects of the clinical features. The results showed that the expression of *HOXA10, HOXA11*, and *HOXA13* and two clinical parameters (M stage and gender) were independent prognostic biomarkers of LSCC outcome. The results of the multivariate Cox proportional hazards regression analysis are exhibited in forest plots in Figure 4.

**Table 1:** Univariate Cox proportional hazards regression analyses of *HOXA* members and clinical features in LSCC.

**Parameter**	**Univariate analysis**		
	**Hazard ratio**	**95% CI**	***P***
Age	1.004	0.969–1.041	0.811
Smoking history	0.659	0.366–1.185	0.164
Alcohol consumption	0.668	0.377–1.1827	0.166
M stage	8.225	1.901–35.594	**0.005**
N stage	1.305	0.744–2.289	0.354
T stage	0.702	0.348–1.4145	0.322
Stage	0.894	0.379–2.108	0.797
Gender	3.367	1.708–6.639	**4.564E−04**
Grade	0.886	0.581–1.351	0.572
*HOXA1* expression	1.384	1.042–1.837	**0.025**
*HOXA2* expression	1.059	0.828–1.356	0.646
*HOXA3* expression	1.238	0.932–1.647	0.140
*HOXA4* expression	1.174	0.857–1.608	0.317
*HOXA5* expression	1.143	0.885–1.477	0.304
*HOXA6* expression	1.105	0.915–1.334	0.299
*HOXA7* expression	1.149	0.93–1.419	0.198
*HOXA9* expression	1.115	0.993–1.252	0.065
*HOXA10* expression	1.379	1.081–1.759	**0.0097**
*HOXA11* expression	1.179	1.000–1.391	**0.0498**
*HOXA13* expression	1.129	0.999–1.277	**0.051**

*Bold means P < 0.05*.

### Correlations Between TIICs and *HOXA* Members

**Figure 5:**
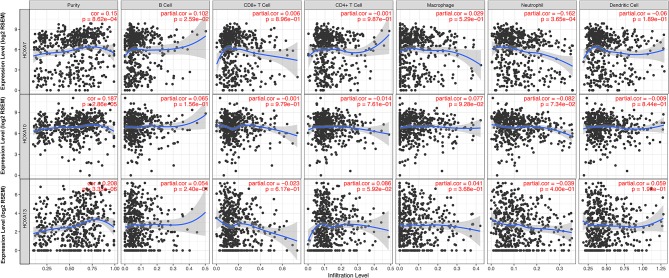
Correlations between tumor infiltrating immune cells (TIICs; B cells, CD4+ T cells, CD8+ T cells, neutrophils, macrophages, and dendritic cells) and *HOXA* members (including *HOXA7, HOXA10*, and *HOXA13*) in LSCC. Tumor purity is shown in the panels on the left.

Considering the increasing evidence on the associations between immunological features and prognosis in cancer, we further explored the correlations between TIICs and *HOXA* members. The TIMER database is a public resource used to explore the associations between certain gene products and immune cells around tumor cells. The first column in Figure 5 shows scatterplots of the expression of *HOXA* members against tumor purity. *HOXA* members with high expression in the microenvironment cells are expected to have a negative association with tumor purity, while *HOXA* members with high expression in tumor cells are expect to have a positive association with tumor purity (17). In accordance with our aforementioned findings, *HOXA7, HOXA10*, and *HOXA13* were highly expressed in LSCC tissues, with positive associations with tumor purity (Figure 5). However, there were no significant correlations between TIICs and *HOXA* members (Figure S2).

### Potential Mechanism Underlying the Effects of Prognostic *HOXA* Members on LSCC Carcinogenesis

**Figure 6:**
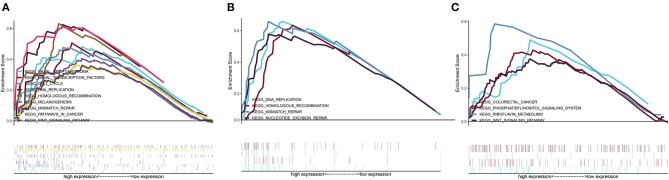
Cancer-related Kyoto Encyclopedia of Genes and Genomes (KEGG) pathways associated with *HOXA10* **(A)**, *HOXA11* **(B)**, and *HOXA13* **(C)** based on a gene set enrichment analysis (GSEA).

A GSEA of differentially expressed *HOXA* members with statistical prognostic value was conducted to evaluate the potential biological mechanism by which differential expression of *HOXA10, HOXA11*, and *HOXA13* affects the carcinogenesis of LSCC. The GSEA indicated that high expression of *HOXA10* was related to “WNT signaling pathway,” “pathway in cancer,” “basal cell carcinoma,” “cell cycle,” “mismatch repair,” and “DNA replication” (Figure 6A), high expression of *HOXA11* was related to “DNA replication,” “mismatch repair,” and “nucleotide excision repair” (Figure 6B) and high expression of *HOXA13* was related to “colorectal cancer” and “WNT signaling pathway” (Figure 6C).

## Discussion

Homeobox genes were first identified in the fruit fly Drosophila (19). A total of 39 *HOX* genes are located on various chromosomes, which are clustered into four clusters, namely *HOXA, HOXB, HOXC* and *HOXD* (4). The genes in these four cluster each encode a 61-amino acid homeodomain, and these genes are key components of master regulatory pathways during normal embryonic development (3). A typical characteristic of the homeodomain is its DNA-binding nature; the proteins function as transcription factors by binding to the promoters of various target genes (20). Increasing evidence has shown that the protein products of *HOXA* genes not only act as transcriptional factors promoting carcinogenesis but also serve as tumor-suppressor factors, based on their aberrant expression patterns in certain organs. Increasing published or public genomic data and multiple online platforms provide us the opportunity for exploring the expression profiles of families of genes in human cancers and their clinical practice value. This study demonstrated the distinct expression profile and methylation profile, prognostic values and biological processes related to *HOXA* members in LSCC.

Previous research has shown that, according to expression data, *HOXA* genes contribute to the development of human cancers. Reverse transcriptase-polymerase chain reaction (RT-PCR) showed that *HOXA7* and *HOXA9* mRNAs were significantly overexpressed in esophageal squamous cell carcinoma tissues compared to non-cancerous surrounding tissues (21), while *HOXA9* was epigenetically downregulated in lung cancer (22). *HOXA13* expression increased in breast cancer (13), whereas it was downregulated in colorectal cancer (10). However, the expression of the entire *HOXA* family in LSCC was not previously comprehensively investigated. This *in silico* study demonstrated the expression profile of *HOXA* members in LSCC and showed that *HOXA2* and *HOXA4* were downregulated in LSCC tissues compared to normal control tissues. In contrast, *HOXA7, HOXA9, HOXA10, HOXA11*, and *HOXA13* were upregulated in LSCC tissues compared to normal control tissues. Unfortunately, no significant differences in the mRNA expression of *HOXA1, HOXA3, HOXA5*, and *HOXA6* were identified in LSCC tissues compared to normal control tissues.

According to the Pearson's correlation between *HOXA* mRNA expression and the methylation level of cg sites in the promoter regions in LSCC, among the seven differentially expressed *HOXA* members (*HOXA2, HOXA4, HOXA7, HOXA9, HOXA10, HOXA11*, and *HOXA13*), most expression levels, particularly regarding *HOXA4* and *HOXA9*, are affected by the methylation level. These results are in accordance with previous findings showing a negative correlation between *HOXA4* methylation and expression in patients with acute myeloid leukemia (23).

Several reports have identified *HOXA* gene signatures in GBM, and high expression of *HOXA9* and *HOXA10* were reported to be predictors of poor outcome in patients with GBM (14, 15). Moreover, it was reported that novel methylation markers in *HOXA9* also served as an independent indicator of prognosis in invasive bladder cancer (24). Additionally, multiple highly expressed *HOXA* members were reported to be significantly correlated with poor overall survival in patients with acute myeloid leukemia (25). In this study, univariate Cox proportional hazards regression analyses were performed to analyze the prognostic values of *HOXA* members in LSCC. In fact, four *HOXA* members were significantly associated with poor clinical outcomes in LSCC (*HOXA1, HOXA10, HOXA11*, and *HOXA13*). Thus, although no significant differential expression of *HOXA1* was found in LSCC, the univariate Cox proportional hazards regression showed that *HOXA1* expression was significantly associated with prognosis. The predictive potential of *HOXA* has also been reported in breast cancer (12). In breast cancer, *HOXA1* knockdown inhibited cell proliferation and increased apoptosis and cell cycle arrest by influencing the aberrant expression of several cell cycle and apoptosis-associated proteins, comprising cyclin D1, B-cell lymphoma 2 (Bcl-2) and Bcl-2-like protein 4 (12). Thus, although *HOXA1* was not differentially expressed in LSCC, the prognostic value of *HOXA1* has been highlighted in various human cancers, including in LSCC. Exploration of the *HOXA1*-related mechanisms is still required.

In hepatocellular carcinoma cells, *HOXA10* knockdown induced cell cycle arrest at the G0/G1 phase and apoptosis by reducing the expression of Cyclin D1 and Survivin (26). Decreased expression of *HOXA10* accelerated the acetylation of p53 (Lys382) and suppressed the transcription of histone deacetylase 1 (HDAC1; a potential deacetylase for p53) to activate p53 transcription (26). Additionally, *HOXA10* might promote cell proliferation by elevating Bcl-2 expression and inhibiting apoptosis in gastric cancer, and high expression of *HOXA10* predicted poor overall survival in gastric cancer patients (27). In this study, we found high expression of *HOXA10* in LSCC tissues. Both univariate and multivariate Cox proportional hazards regression analyses affirmed the prognostic value of *HOXA10* in the prediction of poor outcome in LSCC patients.

Overexpression of *HOXA11* has been observed in ovarian cancer (28), bladder cancer (29), renal cell carcinoma (29) and lung cancer (30), while downregulation of *HOXA11* has been observed in gastric cancer (31) and glioblastoma (32). In glioblastoma, overexpression of *HOXA11* confers a tumor suppressive effect, reduces treatment resistance and contributes to a favorable prognosis (32). However, overexpression of *HOXA11* showed a poor association with overall survival in lung cancer (33). *HOXA11* was significantly downregulated in cisplatin-resistant lung adenocarcinoma cell lines compared with parent cell lines, and *in vitro* experiments showed that overexpression of *HOXA11* increased cisplatin sensitivity by inhibiting Akt/β-catenin signaling (34). Our results showed high expression of *HOXA11* in LSCC, which was associated with unfavorable outcomes in LSCC patients. However, given that there is little relevant research on the topic, the biological and prognostic values of *HOXA11* warrant further intensive investigation. It may be useful to systematically explore the prognostic value of *HOXA11* using meta-analysis.

*HOXA13* is expressed more in normal colons than in malignant colons. Additionally, *HOXA13* was differentially expressed based on location, with higher expression on the left side of the normal colon compared to the right side (10). Differential expression of *HOXA13* was also reported in breast cancer (13), gastric cancer (35), prostate carcinoma (36) and thyroid cancer (37). *HOXA13* knockdown significantly restored the epithelial characteristics and reduced the mesenchymal characteristics of the cancer cells via the transforming growth factor (TGF)-β signaling pathway (35). Moreover, *HOXA13* expression negatively affects cisplatin sensitivity in human esophageal squamous cells and overall survival in patients with esophageal squamous cell carcinoma (38). Our results showed that multiple cancer-associated pathways were identified in LSCC tissues with high expression of *HOXA13*, and high expression of *HOXA13* in LSCC predicted poor overall survival.

## Conclusion

This *in silico* study demonstrated the expression profile of *HOXA* family members in LSCC and the biological and prognostic values of the *HOXA* family in LSCC, providing insights for further investigation of *HOXA* members as potential targets in LSCC.

## Data Availability Statement

The data that support the findings of this study are openly available in The Cancer Genome Atlas (TCGA) program at https://portal.gdc.cancer.gov/.

## Author Contributions

JL and CZ designed the research study and analyzed the data from public database. JL, MY, and CZ were involved in data analysis. CZ was responsible for writing of manuscript. JL and MY contributed to the revised manuscript. All authors reviewed the manuscript.

### Conflict of Interest

The authors declare that the research was conducted in the absence of any commercial or financial relationships that could be construed as a potential conflict of interest.

## Supplementary Material

Figure S1 Pearson's correlation between methylation levels and expression of all differentially expressed HOXA members in LSCC [including *HOXA2* **(A)**, *HOXA4* **(B)***, HOXA7* **(C)**, *HOXA9* **(D)**, *HOXA10* **(E)**, *HOXA11* **(F)**, and *HOXA13* **(G)**].

Figure S2 Correlations between tumor infiltrating immune cells (TIICs; B cells, CD4+ T cells, CD8+ T cells, neutrophils, macrophages, and dendritic cells) and all *HOXA* members in LSCC.
