# Supplementary figures and images for: Expression Profile and Prognostic Values of HOXA Family Members in Laryngeal Squamous Cell Cancer
Source: Front Oncol. 2020 Mar 31;10:368. doi: 10.3389/fonc.2020.00368 (PMC7136465; doi:10.3389/fonc.2020.00368)

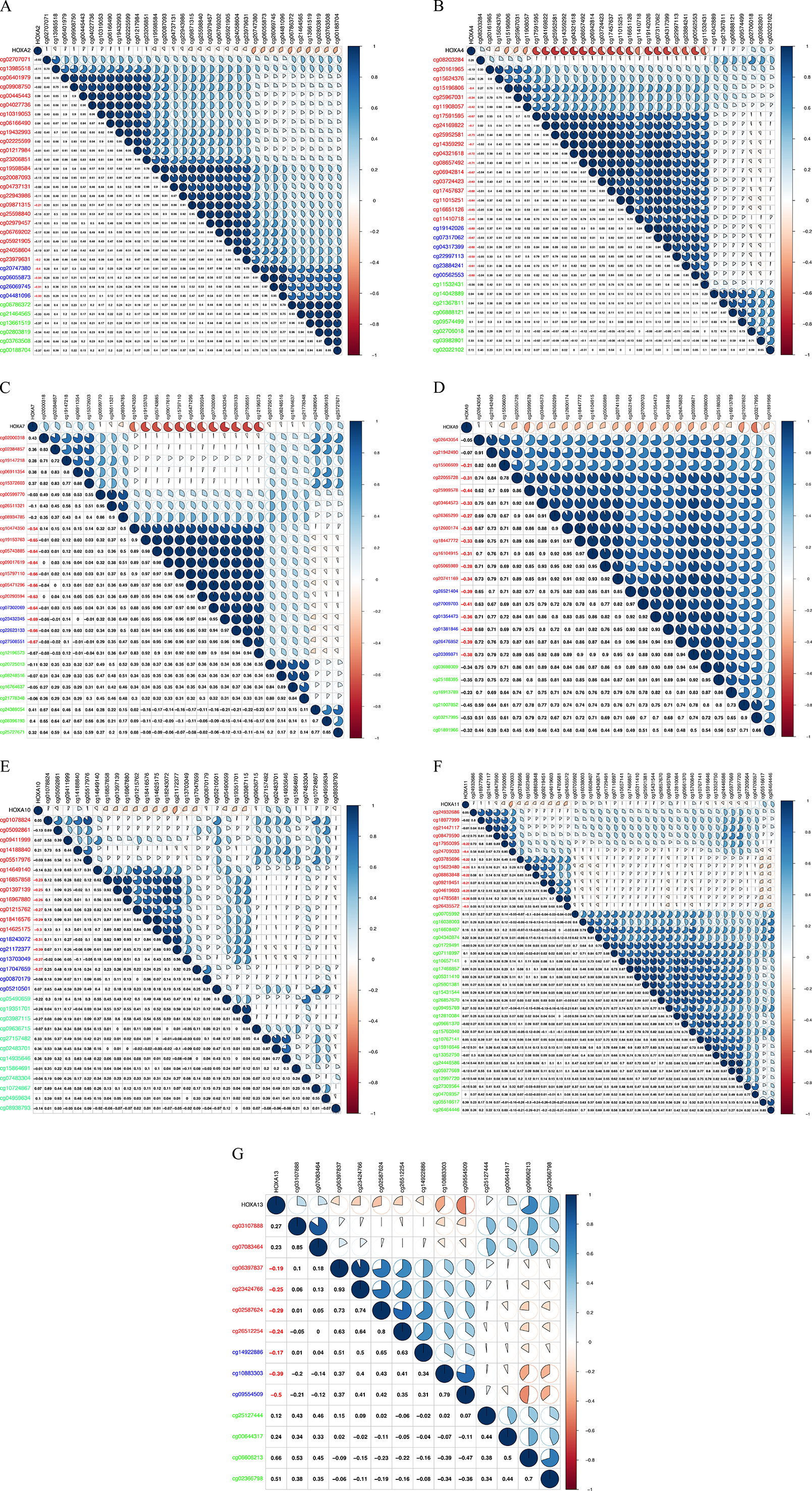

Supplement: Figure S1 — Pearson's correlation between methylation levels and expression of all differentially expressed HOXA members in LSCC [including HOXA2 (A), HOXA4 (B), HOXA7 (C), HOXA9 (D), HOXA10 (E), HOXA11 (F), and HOXA13 (G)]. [file Image_1.TIF]

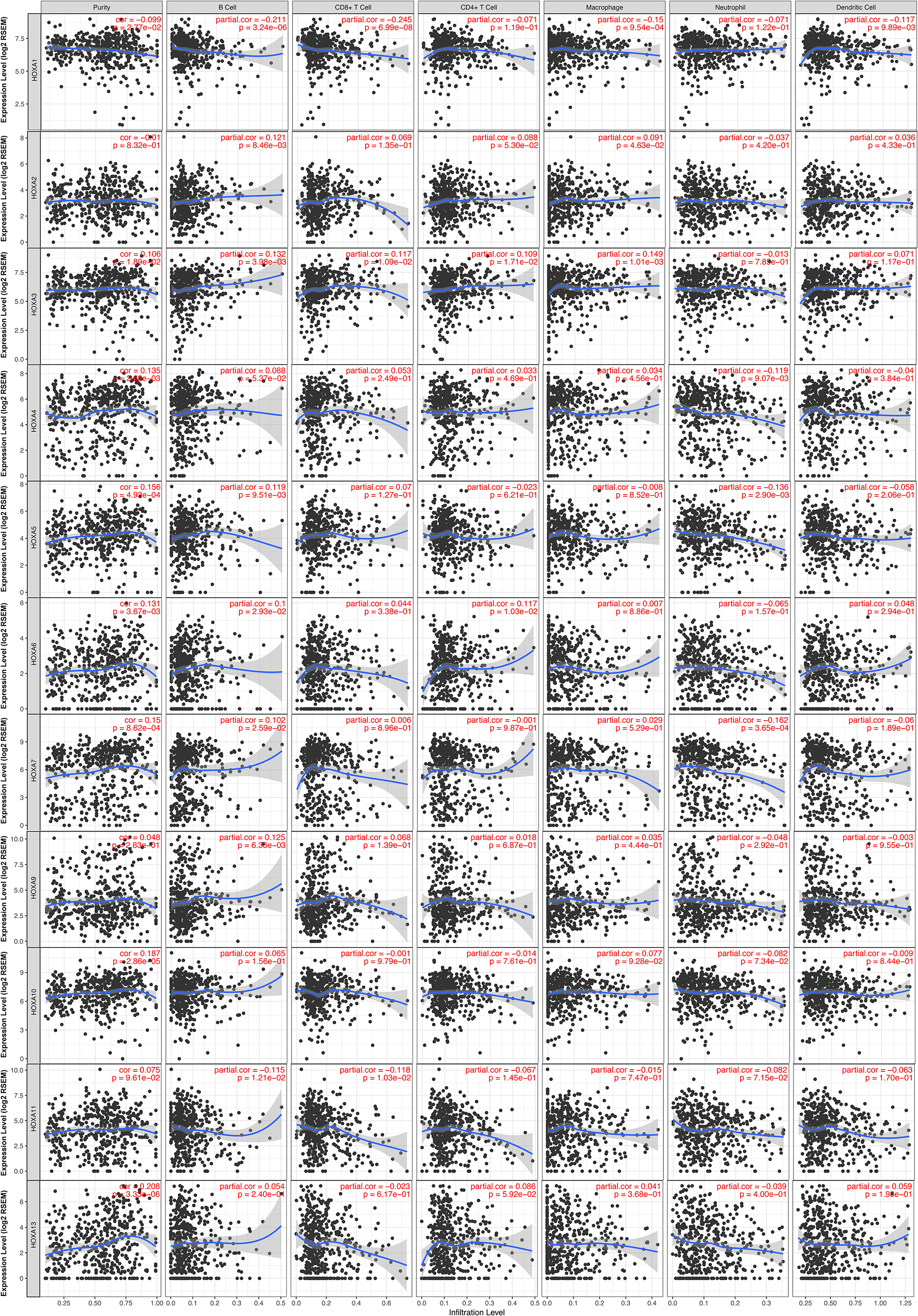

Supplement: Figure S2 — Correlations between tumor infiltrating immune cells (TIICs; B cells, CD4+ T cells, CD8+ T cells, neutrophils, macrophages, and dendritic cells) and all HOXA members in LSCC. [file Image_2.TIF]
